# Role of Exercise During Invasive Hemodynamic Assessment in Symptomatic Adults With a Systemic Right Ventricle

**DOI:** 10.1016/j.jacadv.2022.100023

**Published:** 2022-04-26

**Authors:** C. Charles Jain, Alexander C. Egbe, Yogesh N.V. Reddy, Heidi M. Connolly, Donald J. Hagler, William R. Miranda

The maladaptive response of systemic right ventricles (sRV) to increased afterload is well established, with systolic dysfunction present in most adults. As in those with systemic left ventricles (sLV), individuals with sRV referred to cardiac catheterization typically undergo hemodynamic assessment only at rest. The incremental value of invasive exercise hemodynamics in patients with a sLV and exertional symptoms is unequivocal, as half have normal resting hemodynamics.^1^ The yield of invasive exercise hemodynamics in symptomatic adults with sRVs is unknown.

Twelve patients with transposition of the great arteries (TGA) and sRV (5 complete TGA after atrial switch operation; 7 congenitally corrected TGA [ccTGA]) underwent invasive exercise assessment at Mayo Clinic, Minnesota, between November 2016 and May 2021. Nine individuals underwent supine biking^2^ via right internal jugular venous access, while 3 undergoing femoral access exercised via arm adduction with 5-lb dumbbells. Venous catheterization was performed using 7-F balloon-tipped catheters. Pulmonary artery wedge pressure (PAWP) position was confirmed by fluoroscopy, typical waveforms, and blood oximetry. Simultaneous sRV catheterization was performed in 5. Pressure measurements represent computer-generated averages of ≥5 consecutive beats. Cardiac output was calculated using direct Fick principle in 10 patients and indirect Fick in 2.

Nominal variables are presented as counts (%); continuous variables are presented as median (range). Comparisons between resting and exercise hemodynamics were performed using paired analysis (Wilcoxon signed rank test). The study was approved by the Institutional Review Board; all patients authorized the use of their medical records.

Median age was 47 (35-61) years, and 3 patients (25%) were female (Table 1). Among post-atrial switch patients, 3 underwent the Mustard procedure, 1 the Senning procedure, and 1 the Shumaker modification. Two had ≥moderate superior and/or inferior vena cava baffle obstruction; one had ≥moderate pulmonary venous baffle obstruction. Among those with ccTGA, 3 had normally functioning pulmonary conduits and 3 had normal mechanical systemic tricuspid prostheses.

**Table 1:** Patient Characteristics and Resting and Exercise Hemodynamics

	Case 1	Case 2	Case 3	Case 4	Case 5	Case 6	Case 7	Case 8	Case 9	Case 10	Case 11	Case 12
Anatomy	TGA	TGA	TGA	TGA	TGA	ccTGA	ccTGA	ccTGA	ccTGA	ccTGA	ccTGA	ccTGA
Patient characteristics												
Age (y)	35	40	44	39	37	57	57	49	54	41	49	61
NYHA functional class	II	III	III	II	III	III	III	II	III	III	II	III
Symptoms	A	A, D, L	A, D, L	D, F, L	D, F	D, F	D, F	A, D, F	D, F	D, L	D, F	D, F
Rhythm	SR, VS	SR, VS	SR, VS	SR, VS	SR, VS	AF, VP	SR, VS	SR, VS	AF, VP	AF, VP	AF, VP	SR, VP
Creatinine (mg/dL)	0.80	0.80	0.94	1.12	1.26	8.56	1.29	1.37	1.72	1.06	1.45	1.44
NT-proBNP (pg/mL)	403	347	1,297	188	-	53,255	981	935	2,193	667	-	3,187
Peak VO_2_ (L/min/kg) (% predicted)	17.5 (51%)	14.5 (35%)	29.4 (65%)	12.1 (29%)	23.7 (55%)	-	12.5 (38%)	14.2 (39%)	13.0 (52%)	16.7 (40%)	12.5 (34%)	10.4 (33%)
sRV EF (%)	42.5	17.5	30	45	45	25	30	35	35	20	15	20
Systemic TR	1	1	2	1	2	3	3	5	4	1p	1p	1p

Catheterization	R	E	R	E	R	E	R	E^a^	R	E	R	E^a^	R	E^a^	R	E	R	E	R	E	R	E^b^	R	E
HR (beats/min)	69	88	75	87	70	135	93	94	65	108	68	71	65	108	60	92	69	70	50	50	60	60	92	124
Arterial SP (mmHg)	104	135	114	122	138	155	118	124	118	125	76	97	94	124	133	126	84	94	100	134	72	90	81	101
Systemic venous pressure (mmHg)	7	15	12	19	8	14	21	24	11	18	-	-	2	4	3	16	13	29	9	23	12	23	8	30
mPAP (mmHg)	20	35	32	46	28	52	32	39	17	26	25	33	31	54	17	34	21	36	30	46	51	50	36	66
PAWP (mmHg)	13	26	23	37	21	47	21	31	10	27	9	15	13	26	10	27	15	28	17	34	20	22	24	44
PAWP-V (mmHg)	23	49	34	64	38	71	35	39	16	37	12	18	23	36	11	36	22	42	29	51	26	28	33	59
sRV EDP (mmHg)	11	24	21	29	9	28^a^	15	18	-	-	8	13	10	17	-	-	-	-	-	-	-	-	-	-
CI (L/min/m^2^)	2.7	-	2.7	-	1.4	3.7	2.3	-	1.6	5.2	1.9	-	2.6	-	1.5	3.0	2.1	2.1	2.3	2.4	1.7	1.7	1.9	3.2
ΔPAWP/ΔCO	-		-		7.2		-		1.5		-		-		6.2		↑		↑		↑		7.6	
PVR (WU)	1.0	-	1.7	-	3.1	0.9	2.1	-	2.1	-	5.1	-	3.6	-	2.6	1.3	1.6	1.6	2.5	2.2	9.5	8.5	3.0	3.3

↑ indicates elevated ratio but not reported given minimal change in cardiac output.

A = angina; AF = atrial fibrillation/flutter; ccTGA = congenitally corrected transposition of the great arteries; CI = cardiac index; D = dyspnea on exertion; E = exercise; EDP = end-diastolic pressure; EF = ejection fraction; F = fatigue; HR = heart rate; L = leg edema; mPAP = mean pulmonary artery pressure; NT-proBNP = N-terminal pro brain natriuretic peptide; NYHA = New York Heart Association; PAWP = mean pulmonary artery wedge pressure; PAWP-V = pulmonary artery wedge pressure V wave; PVR = pulmonary vascular resistance; R = rest; SP = systolic pressure; SR = sinus rhythm; sRV = systemic right ventricle; TGA = complete transposition of the great arteries status after atrial switch; TR = tricuspid regurgitation; VP = ventricular pacing; VS = ventricular sensing; 1 = mild; 1p = mild prosthetic; 2 = mild-moderate; 3 = moderate; 4 = moderate-severe; 5 = severe.

a Obtained during arm exercise.

b Submaximal exercise.

All patients had exertional symptoms (dyspnea in 11, angina in 4), with 8 (67%) having New York Heart Association functional class III. Median peak oxygen consumption (VO_2_) during noninvasive cardiopulmonary exercise test was 14.2 (10.4-29.4) l/min.kg (39% [29%-65%] of predicted). Echocardiography revealed ≥moderate sRV enlargement in 11 patients (92%) and estimated sRV ejection fraction 30% (15%-45%); ≥moderate systemic atrioventricular valve regurgitation was present in 4 (all had ccTGA). Severe subpulmonary atrioventricular valve regurgitation and moderate pulmonary regurgitation were present in 1 individual each with ≥moderate LV outflow tract obstruction seen in 2.

Exercise catheterization was successfully performed without procedural complications in all individuals. Resting PAWP was 16 (9-24) mmHg, being ≤15 mmHg in 5 patients (42%). During exercise, PAWP significantly increased (28 [15-47] mmHg; *P* = 0.005), rising above 25 mmHg in all but 2 patients. Prominent PAWP v-waves (44 [18-71] mmHg) were seen during exercise, particularly in patients who underwent atrial switch. Those with a change of >20 bpm in heart rate (n = 5) had significantly higher exercise ΔPAWP (19 [17-25] vs 13 [7-14] mmHg; *P* = 0.01).

Similarly, systemic venous pressure (8 [2-13] to 16 [4-29]) and pulmonary artery mean pressure (28 [17-32] to 46 [34-54] mmHg) significantly increased during exercise (*P* = 0.02 for both). Following supine cycling, 1 patient underwent exercise with arm weights after femoral arterial access was obtained; PAWP was 33 (v-wave 55) mmHg during arm exercise and 47 (v-wave 71) mmHg while cycling.

Resting cardiac index was 2.0 (1.4-2.7) L/min/m^2^. Seven patients had continuous VO_2_ measurements during cycling exercise; 4 had an increase in CO of ≥80% from what was predicted,^2^ whereas in 3, the cardiac output failed to rise. For those who augmented CO, the ΔPAWP/ΔCO ratios were 1.5, 6.2, 7.2, and 7.6 mmHg/L/min.

We acknowledge the small, heterogeneous sample, an inherent limitation of studies in TGA, but our initial experience with invasive exercise hemodynamics in patients with sRV raises several important points: 1) despite anatomical differences compared with sLVs, exercise catheterization is feasible in patients with sRV; 2) in a relatively old and highly symptomatic population, resting PAWP was normal in nearly 50% of the cohort; 3) exercise PAWP was elevated in most patients; 4) ΔPAWP/Δcardiac output ratios were markedly abnormal and higher than expected for acquired disease^1^ even in those with normal CO reserve, indicating significantly impaired pulmonary venous atrial and sRV compliance.

Despite most adults with sRVs having systolic dysfunction, the benefit of goal-directed medical therapy in these patients is unclear. Thus, clinicians are left with tailoring therapy to individual patients. This challenge is deepened by the lack of data regarding noninvasive assessment of filling pressures and the ubiquitous impaired exercise performance on exercise testing. Mirroring findings in patients with sLV, it was only with exercise that underlying hemodynamics were fully revealed and the magnitude of exertional symptoms and functional limitation explained.

We have now incorporated exercise at the time of catheterization as an additional tool in the clinical evaluation and management of these complex patients. For example, 1 patient had severe dynamic subpulmonic stenosis with only mild elevation of PAWP during exercise, thus more diuresis was felt to be detrimental. In another patient, marked hemodynamic abnormalities with exercise (PAWP >40 mmHg) prompted transplant evaluation. We hope our preliminary results stimulate further studies on invasive exercise hemodynamics in sRVs. This will also allow better understanding of the differences in hemodynamics between adults who underwent atrial switch compared with ccTGA and perhaps individualize therapy.

Noteworthy, several patients reported angina in the absence of coronary atherosclerosis. Supply-demand mismatch has been discussed in those with sRV, and the observed elevated end-diastolic sRV pressures during exercise in our study support that theory and could impact management by avoiding antianginals and prioritizing heart failure management.

In summary, exercise cardiac catheterization is feasible in patients with sRVs, and similar to patients with sLVs, it might provide incremental diagnostic information in the assessment of these individuals.
